# *Dhdds* T206A and *Dhdds* K42E knock-in mouse models of retinitis pigmentosa 59 are phenotypically similar

**DOI:** 10.1242/dmm.052243

**Published:** 2025-08-01

**Authors:** Mai N. Nguyen, Dibyendu Chakraborty, Jeffrey Messinger, Timothy W. Kraft, David M. Sherry, Steven J. Fliesler, Steven J. Pittler

**Affiliations:** ^1^ Department of Optometry and Vision Science, Vision Science Research Center, School of Optometry, University of Alabama at Birmingham, Birmingham, AL 35294, USA; ^2^ Departments of Cell Biology, Neurosurgery, and Pharmaceutical Sciences, University of Oklahoma Health Sciences Center, Oklahoma City, OK 73104, USA; ^3^ Research Service, VA Western New York Healthcare System, Buffalo, NY 14215, USA; ^4^ Departments of Ophthalmology and Biochemistry and Neuroscience Graduate Program, Jacobs School of Medicine and Biomedical Sciences, The State University of New York, University at Buffalo, Buffalo, NY 14203, USA

**Keywords:** Inherited retinal degeneration, Retinitis pigmentosa, RP59, Mouse models, Glycosylation, Dolichol

## Abstract

Dehydrodolichyl diphosphate synthase complex subunit (DHDDS) is required for protein glycosylation in eukaryotes, and variants. Surprisingly, three variant alleles (K42E/K42E, T206A/K42E and R98W/K42E) have been reported to cause retinitis pigmentosa 59 (RP59). Because T206A only has been reported to occur heterozygously with K42E, we generated homozygous and hererozygous mutants – i.e. T206A/T206A and T206A/K42E, respectively – in mice to assess the effect of the T206A allele. By postnatal age of 12 month (PN 12-mo), T206A/T206A and T206A/K42E mice exhibited reduction of inner nuclear layer thickness as observed in K42E/K42E mice. Electroretinography (ERG) revealed a reduction in b-waves, but spared reduction in a-wave amplitudes. By PN 3-mo, ERG c- and d-waves were significantly attenuated in all phenotypes. Consistent with a reduction in inner nuclear layer thickness as seen by using optical coherence tomography (OCT), cell loss observed by histology, as well as bipolar and amacrine cell densities were reduced in all *Dhdds* mutant phenotypes compared to those of PN 8-12 mo age-matched controls. These results indicated that the *DHDDS* T206A allele, like the K42E allele, causes retinal disease, probably through a common pathobiological mechanism. We propose that the physiological basis of retinal dysfunction in RP59 involves defective photoreceptor to bipolar cell synaptic transmission with concomitant bipolar/amacrine cell degeneration.

## Teaser

**Summary:** *DHDDS* in humans encodes a protein required for a fundamental biological modification of protein that is required for viability, yet certain *DHDDS* gene variants only lead to visual dysfunction. We have generated mouse models of these gene variants to determine the mechanistic basis of the selective ocular disease.

## INTRODUCTION

In the dolichol synthesis pathway, two subunits of dehydrodolichyl diphosphate synthase (DHDDS) (OMIM #608172) partner with two subunits of NUS1 dehydrodolichyl diphosphate synthase (NgBR, officially known as NUS1) (OMIM #610463) to form the enzyme complex *cis*-prenyltransferase (Bar-El et al., 2020; Brasher et al., 2015; Edani et al., 2020; Sato et al., 1999). This enzyme complex catalyzes the sequential repeated addition of the five-carbon building unit isopentenyl diphosphate (IPP) to farnesyl diphosphate (FPP) to form polyprenyl diphosphates that, ultimately, are converted to dolichol (Dol) units of varying chain lengths, typically comprising 18 to 20 Dols (Dol-18 to Dol-20, respectively). Recently, the current information regarding exactly how Dol is formed has been challenged by findings that implicate the formation of aldehydes (polyprenal and dolichal) as obligate intermediates in the synthesis of Dol (Wilson et al., 2024). Dol is required for protein glycosylation (Brasher et al., 2015; Doucey et al., 1998; Kornfeld and Kornfeld, 1985; Sato et al., 1999; Welti, 2013), and its phosphorylated derivative serves as a lipid-soluble carrier for sugar nucleotides and for the formation of oligosaccharide chains that are utilized for protein glycosylation (Aebi, 2013; Cherepanova et al., 2016). Disorders arising from defects in any of the genes encoding the more than 30 enzymes in the dolichol and protein glycosylation pathways have been grouped together as a family, and are termed congenital disorders of glycosylation (CDG) (Cantagrel and Lefeber, 2011; Eklund and Freeze, 2006). Absence of DHDDS is embryonic lethal (Brandwine et al., 2021), whereas more subtle variants in the enzyme lead to potentially fatal downstream effects within the glycosylation pathway (Cantagrel and Lefeber, 2011; Hemming, 1992; Sabry et al., 2016) or to moderate to severe brain disease – including epileptic encephalopathies, myoclonus ataxia or intellectual deficit disorder (IDD), Parkison-like symptoms and neurodevelopmental disorder (Galosi et al., 2021; Kim et al., 2021; Piccolo et al., 2021). Three amino acid variants in the *DHDDS* gene – i.e. lysine to glutamate at amino acid (aa) position 42 (K42E), threonine to alanine at aa position 206 (T206A) and arginine to tryptophan at aa position 98 (R98W) have been reported to cause a recessive form of retinitis pigmentosa called retinitis pigmentosa 59 (RP59) (OMIM #613861) (Fliesler et al., 2022; Kimchi et al., 2018; Sabry et al., 2016; Venturini et al., 2015; Zelinger et al., 2011; Züchner et al., 2011). Surprisingly, this hereditary retinal disease is non-syndromic – i.e. associated pathologies are restricted to the retina, without any obvious systemic involvement – despite the fact that DHDDS is required for dolichol synthesis and, in turn, for protein glycosylation in every cell type and tissue throughout the body.

Retinitis pigmentosa (RP) comprises a heterogeneous group of individually rare genetic disorders of the retina that cause impaired vision and, ultimately, blindness in about 1:4000 individuals worldwide (Ali et al., 2017). The classic form of the disease first disrupts the rod photoreceptors, primarily causing reduced night vision (nyctalopia). Peripheral vision is initially affected, causing tunnel vision but, as the disease progresses with time, central vision (mediated by cone photoreceptors) also diminishes and can, eventually, result in total blindness (Fahim, 2023). Variants in more than 70 genes have been linked to classic RP and over 300 genes are involved in inherited retinal diseases (https://sph.uth.edu/retnet/).

We have previously reported on a novel murine knock-in model of RP59 (*Dhdds*^K42E/K42E^) that neither exhibits overt signs of photoreceptor degeneration nor altered glycosylation up to postnatal age of 12 month (PN 12-mo). There was, however, marked increase in immunostaining for GFAP (glial fibrillary acidic protein) (Ramachandra Rao et al., 2020), indicative of a reactive Müller cell glial response (gliosis) to disturbance in the environment of the retina. In-depth examination revealed reduced thickness of the inner nuclear layer (INL), retraction of photoreceptor terminals and neuronal sprouting of horizontal and bipolar cell processes into the outer nuclear layer (ONL), which are all typical for various forms of photoreceptor degeneration (Nguyen et al., 2023). Physiologically, these mutant mice exhibited negative b-wave ERG (i.e. ERG amplitudes below baseline) waveforms. Analysis of isoprenoid lipids showed shortened dolichol species compared to those of wild-type (WT) control mice, with the mutants exhibiting a shift to Dol-17/Dol-18 species compared to Dol-18/Dol-19 as the dominant species in WT mice, similar to analysis of Dol species in bodily fluids of persons diagnosed with RP59 (Wen et al., 2013). Surprisingly, although RP59 is classified as a CDG, there was no evidence of compromised global protein *N*-glycosylation in the RP59 mouse model (Ramachandra Rao et al., 2020).

In this current study, we characterized another *DHDDS* allele variant – T206A – that is only found heterozygously with the K42E allele in patients with RP59 (Wen et al., 2013). We created a novel *Dhdds*^T206A/K42E^ (T206A/K42E) compound heterozygous mouse line to assess pathogenicity and compare the murine and human phenotypes associated with these *DHDDS* mutations. In addition, we created and characterized homozygous *Dhdds*^T206A/T206A^ (T206A/T206A) and heterozygous *Dhdds*^T206A/WT^ (T206A/WT) mice to assess the pathogenicity of the T206A allele independently to that of the K42E allele. Our results showed that the T206A mutant allele itself is pathogenic, and that the T206A and K42E alleles cause similar phenotypes that are likely to originate from the same disease mechanism.

## RESULTS

### Creation of a T206A DHDDS knock-in mouse line by using CRISPR/Cas9 technology

**Fig. 1.**
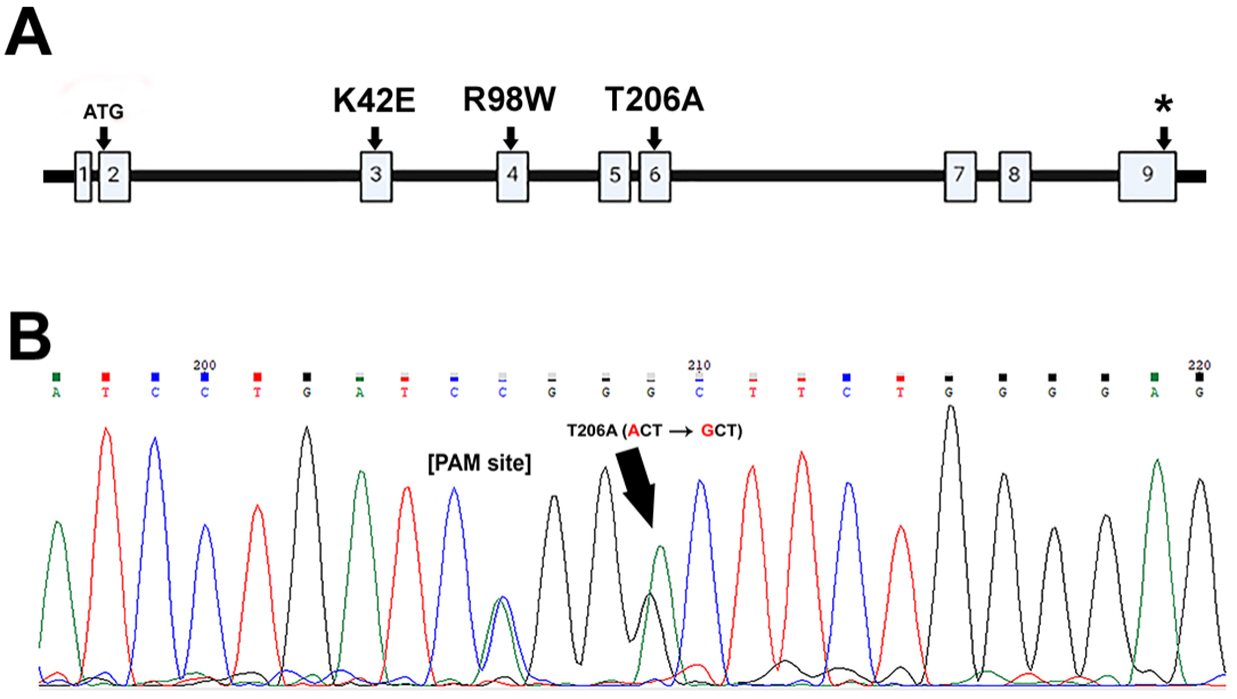
**Verification of T206A knock-in mutation.** (A) Simple schematic representation of the DHDDS gene, showing its nine exons, with the adenine-thymine-guanine (ATG) start codon in exon 2, Lys42Glu (K42E) is contained within exon 3, Arg98Trp (R98W) is contained within exon 4, Thr206Ala (T206A) is contained within exon 6; the stop codon in exon 9 is indicated by an asterisk (*). K42 and T206 flank the catalytic binding sites, including a single Mg^2+^ ion per subunit and multiple isopentenyl units (Bar-El et al., 2020). (B) DNA sequence of a tail sample from an F0 founder mouse, showing the adenine-to-guanine (A-to-G) T206A variant (black arrow) as well as a (cytosine-to-adenine) C-to-A silent heterozygous polymorphism to remove the CRISPR-related PAM recognition site.

DHDDS is an ubiquitous protein encoded by 333 codons distributed across eight exons with one additional noncoding 5′-exon (Fig. 1). The K42E mutation is within exon 3, R98W is within exon 4 and T206A is within exon 6 (Fig. 1A). Functional domains of the DHDDS protein are encoded within exons 3–6, including a co-factor binding site for Mg^2+^ (one Mg^2+^ ion bound to each subunit) and multiple isopentenyl diphosphate binding sites. In our study, the T206A knock-in mouse line was generated using modified CRISPR/Cas9-based technology. The T206A knock-in mutation was confirmed in T206A/WT mice by mouse tail DNA sequence analysis of a PCR product amplified from the *Dhdds* locus (arrow, Fig. 1B), showing the nucleotide change from adenine to guanine (A-to-G). In addition, a silent cytosine to adenine (C-to-A) polymorphism was included in the construct to remove the PAM site sequence required by CRISPR for DNA cleavage (Fig. 1B).

### Histological characteristics of the retinal degeneration in DHDDS mutant mice

**Fig. 2.**
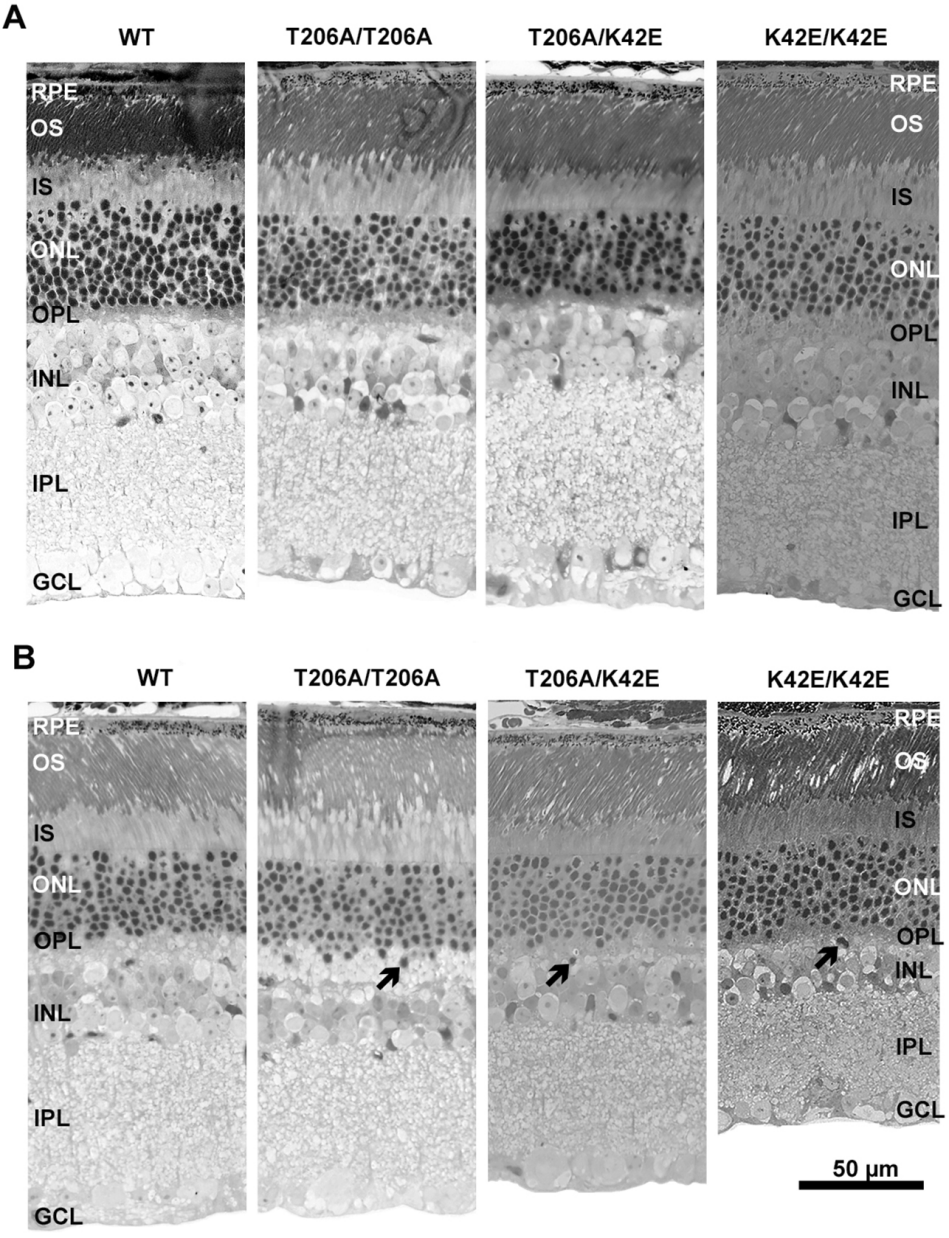
**Retinal histology.** (A,B) Light micrographs of mouse retina obtained from WT, T206A/T206A, T206A/K42E or K42E/K42E mice are shown for younger (PN≤6-mo) (A) and older animals (PN≥12-mo) (B) animals. Previously published K42E/K42E micrographs (Nguyen et al., 2023) were added for comparison. Figure adapted from Nguyen et al., 2023 under the terms of the CC-BY 4.0 license. Thickness of the INL is reduced in mutant strains. Arrows indicate ectopic migration of cells from the ONL into the OPL and INL. RPE, retinal pigment epithelium; OS, outer segment layer; IS, inner segment layer; ONL, outer nuclear layer; OPL, outer plexiform layer; INL, inner nuclear layer; IPL, inner plexiform layer; GCL; ganglion cell layer. Scale bar: 50 µm, all panels.

Transverse epoxy sections of retinas from PN≤6-mo mice of genotypes WT, T206A/T206A, T206A/K42E and K42E/K42E all show normal retinal layer stratification (Fig. 2A). Well-formed outer and inner plexiform layers (OPL and IPL, respectively) were present in all genotypes. Pathologic changes in retinal organization became apparent in T206A/T206A, T206A/K42E and K42E/K42E mice at older ages (PN≥12-mo; Fig. 2B). Ectopic rod cell bodies invading the OPL were present in T206A/T206A, T206A/K42E and K42E/K42E mice (arrows, Fig. 2B). Retinal thinning was also evident, particularly in the INL and IPL of T206A/K42E and K42E/K42E mice, indicating loss of inner retinal cells. Inner retinal changes were less pronounced in compound heterozygous T206A/K42E mice.

### The T206A *Dhdds* variant causes thinning of the retina

**Fig. 3.**
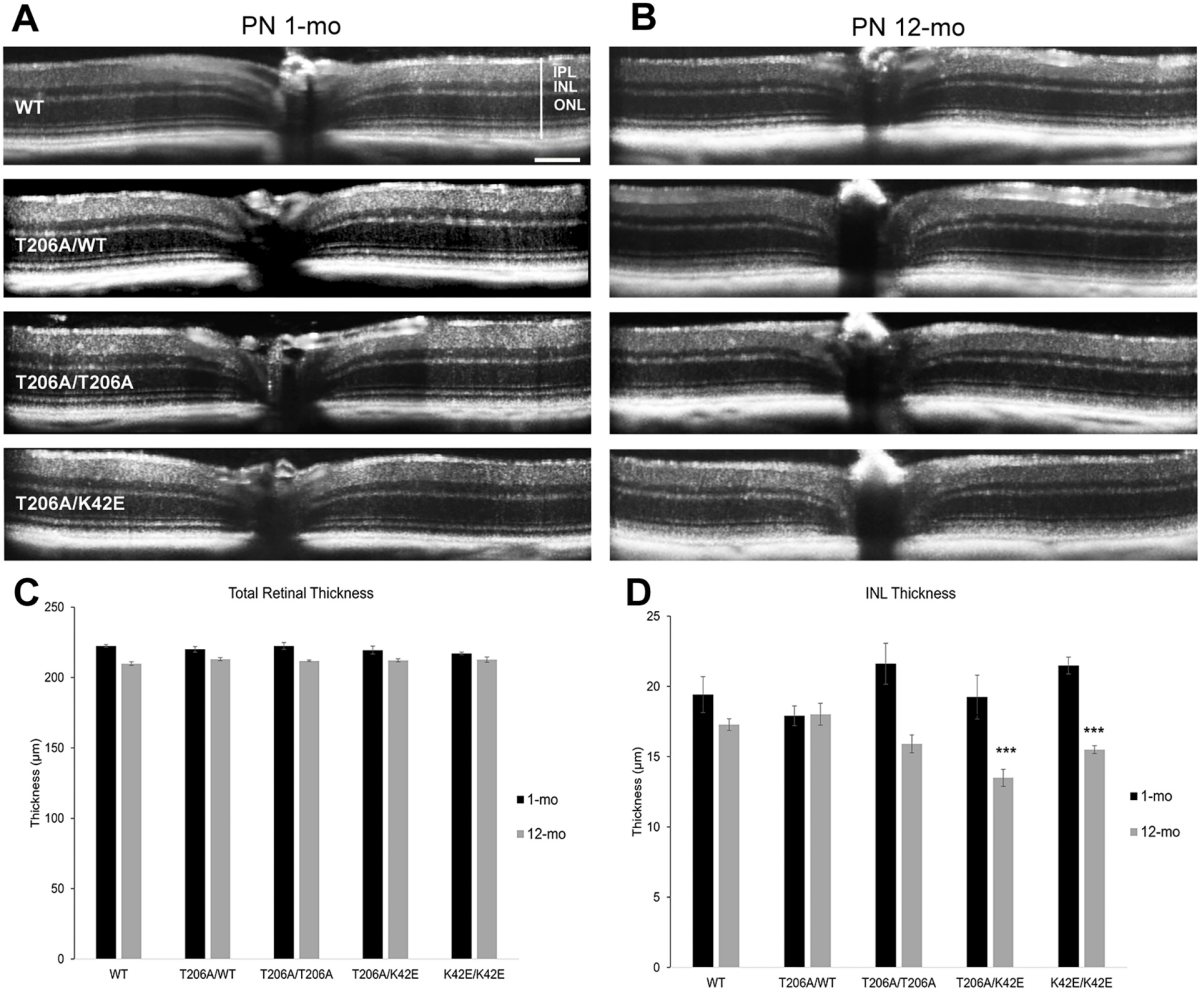
**Measurements of total retinal thickness and INL thickness obtained using SD-OCT.** (A,B) Representative SD-OCT images obtained from WT, T206A/WT, T206A/T206A or T206A/K42E at PN 1-mo (A) and PN 12-mo (B). (C,D) Analysis of the total retinal thickness (indicated by the vertical white line shown in the top left image) (C) and thickness of the inner nuclear layer (INL) (D). Plotted are the averaged data obtained from mice at PN 1-mo (black) and PN 12-mo (grey). Previously published data obtained from K42E/K42E mice (see Nguyen et al., 2023) have been included for reference in panels C and D. Figure adapted from Nguyen et al., 2023 under the terms of the CC-BY 4.0 license. IPL, inner plexiform; ONL, outer nuclear layer. Scale bar: 0.1 µm for all panels. Statistical significance: ***P≤0.0001; *n*=4 (WT); *n*=3 (T206A/WT, T206A/T206A and T206A/K42E).

To further characterize the effects of the T206A variant on retinal structure, we performed spectral domain optical coherence tomography (SD-OCT) to quantify changes in total retinal thickness, and thickness of the ONL and INL. Representative SD-OCT images of WT, T206A/WT, T206A/T206A and T206A/K42E mice at PN 1-mo (Fig. 3A) and PN 12-mo (Fig. 3B) are presented in Fig. 3. Average total retinal thickness for WT, T206A/WT, T206A/T206A and T206A/K42E mice was analyzed at PN 1-mo and PN 12-mo (Fig. 3C). There were no overt differences among the T206A/WT, T206A/T206A or T206A/K42E mice compared to their age-matched WT controls at either time point. However, we observed significant decreases in total retinal thickness at PN 12-mo compared to PN 1-mo: WT had a 5% (*P*≤0.001) reduction, T206A/WT had a 3% reduction (*P*≤0.05), T206A/T206A showed a 5% reduction (*P*≤0.01) and T206A/K42E had a 3% reduction (*P*≤0.05) in total retinal thickness. Previously published total retinal thickness measurements for K42E/K42E have been included for comparison (Fig. 3C) (Nguyen et al., 2023).

Measurements of INL thickness were compared and graphed in Fig. 3D. At PN 1-mo, none of the mutant mouse strains showed significant differences in INL thickness when compared to WT mice. Thickness of the INL did not differ at PN 1-mo or PN 12-mo between WT and T206A/WT mice. In contrast, both T206A/T206A and T206A/K42E mice showed significantly reduced INL thickness at PN 12-mo compared to that at PN 1-mo (reduction of 26% and 30% respectively; *P*≤0.01 for both). In addition, at PN 12-mo, the INL thickness in retinas of T206A/K42E mice was reduced by 21.9% compared to those of age-matched WT mice (*P*≤0.001). Included for reference Fig. 3D includes previously published INL data obtained from K42E/K42E mice, which show a significant reduction in INL thickness at PN 1-mo compared with PN 12-mo (Nguyen et al., 2023). Comparison of ONL thickness at PN 1-mo and PN 12-mo, and across the 12-mo time frame, showed no significant differences (PN 1-mo WT 55.4±1.2, T206A/WT 53.8±1.3, T206A/T206A 55.4±0.9, T206A/K42E 56.3±0.5; PN 12-mo WT 52.8±1.0, T206A/WT 54.7±0.4, T206A/T206A 56.1±1.0, and T206A/K42E 54.4±1.1 μm, mean; s.e.m.).

### The T206A mutation in Dhdds impairs synaptic transmission to bipolar cells

**Fig. 4.**
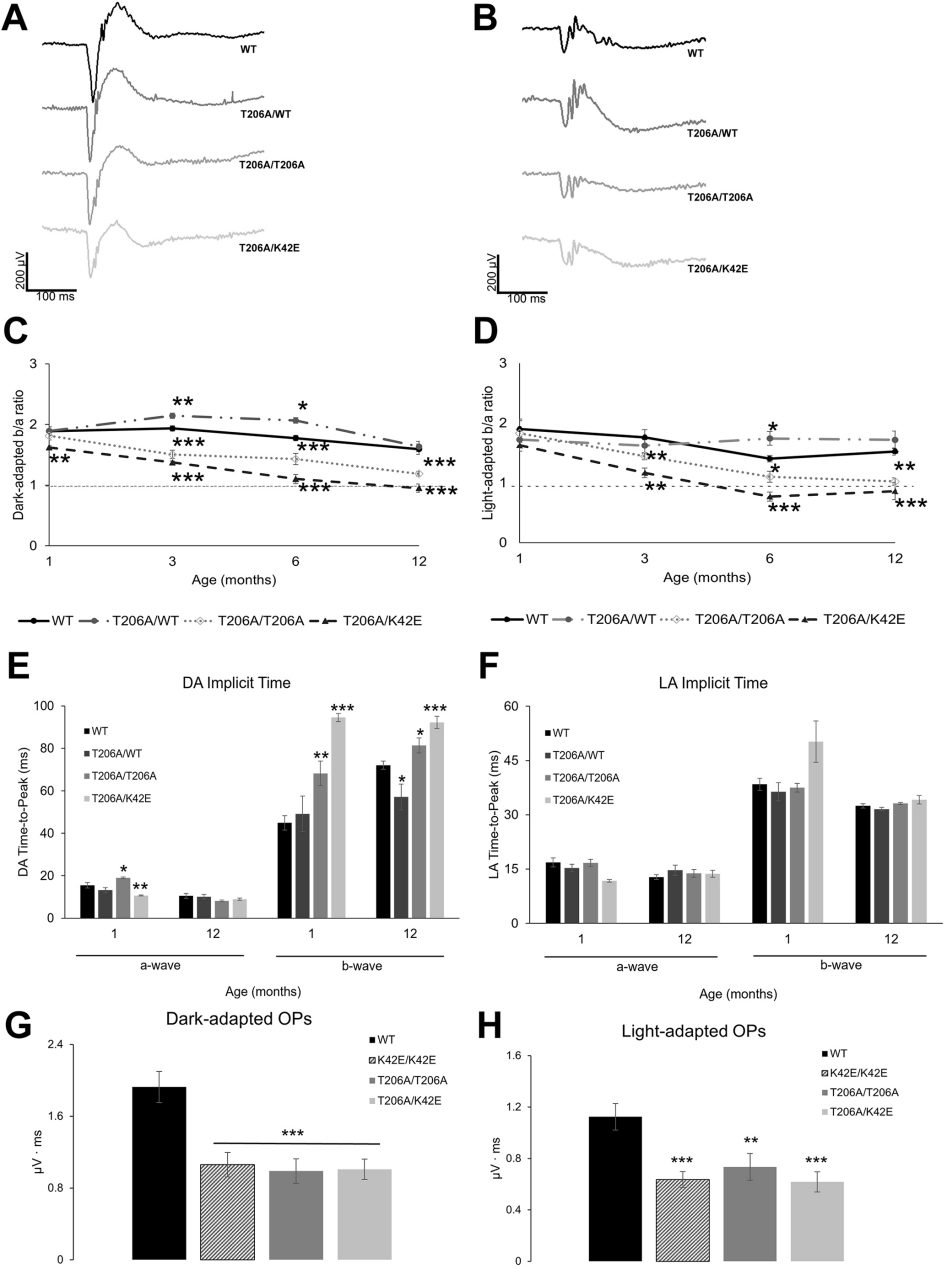
**ERG b/a-wave amplitude ratios.** (A,B) Representative graphs showing dark-adapted (DA) (A) and light-adapted (LA) (B) waveforms obtained from WT. T206A/T206A or T206A/K42E mice at PN 6-mo. (C,D) Plotted b/a ratios taken at PN 1-, 3-, 6- and 12-mo time points under DA (C) and LA (D) conditions are shown for mice as in A,B. All mutant mouse b/a ratio comparisons within each time point were made in relation to WT values. WT (solid black line), T206A/WT (dashed/dotted grey line), T206A/T206A (dotted grey line), T206A/K42E (dashed grey line). The negative b-wave ERG threshold is indicated by the straight horizontal dashed line at b/a=1. (E,F) Implicit time (time-to-peak) measurements for DA (E) and LA (F) a- and b-waves of for mice as in A,B at PN 1- and PN 12-mo are compared to those for WT. (G,H) Oscillatory potential (OP) measurements for DA (G) and LA (H) waveforms obtained from K42E/K42E, T206A/T206A and T206A/K42E mice at PN 12-mo compared to those of WT mice. Statistical significance: *P<0.05, **P<0.01, and ***P≤0.001. Animal numbers varied from *n*=6–14.

Representative graphs of dark-adapted (DA) and light-adapted (LA) ERG waveforms taken at PN 6-mo for WT, T206A/WT, T206A/T206A and T206A/K42E mice are shown in Fig. 4A and B, respectively. The DA photoresponses represent rod-driven (scotopic) responses, while the LA photoresponses represent cone-driven (photopic) responses. While there is some reduction apparent at later time-points (PN 12-mo) in the a-waves of T206A/T206A mice (DA PN 12-mo reduced by 37%, *P=*0.004) and T206A/K42E mice (DA PN 12-mo by 30%, *P=*0.02), the most robust reduction was observed in b-waves in both DA and LA responses (Table S1). For DA responses, the b-wave was greatly reduced by PN 3-mo in T206A/T206A mice (35%, *P=*0.008) and in T206A/K42E mice (40%, *P=*0.004). For LA responses, b-wave reduction was apparent in T206A/K42E mice (30%, *P=*0.04) at PN 3-mo, and in both genotypes at PN 6-mo (≥42%, *P*≤0.01). For comparison across genotypes, b-wave-to-a-wave (b/a) amplitude ratios (Table S1) were averaged and plotted under DA (Fig. 4C) and LA (Fig. 4D) conditions. Under DA conditions, T206A/WT and T206A/T206A mice showed no difference in b/a ratios compared to those of WT at PN 1-mo. In contrast, under DA conditions at PN 1-mo, T206A/K42E mice showed a significant decrease in b/a ratios (1.6±0.03, 15.8%, *P*≤0.01) compared to those of WT (1.9±0.1). From PN 3-mo to PN 12-mo, b/a ratios for T206A/T206A and T206A/K42E mice were progressively reduced compared to those of their age-matched WT counterparts (*P*≤0.001 for both genotypes at all time points). By PN 12-mo, the T206A/K42E b/a ratio fell to 1.0 – i.e. the threshold for negative b-wave as defined by Tanimoto et al. (2015) – equal to a 34% decline (*P*≤0.001). Interestingly, the b/a ratio for T206A/WT mice was significantly higher than that for WT mice at PN 3- and 6-mo (*P*≤0.05).

Under LA conditions, the b/a ratios for T206A/T206A and T206A/K42E mice did not differ from that for WT mice until PN 3-mo, when they were reduced by 16% and 30%, respectively (*P*≤0.01). At PN 6-mo, the b/a ratio for T206A/K42E mice dropped below the negative b-wave ERG (hereafter referred to as negative ERG) threshold of b/a=1 (by 36%, *P*=0.02) and stayed below that threshold at PN 12-mo (reduced by 43%, *P*≤0.001). Unlike the K42E/K42E mice (Nguyen et al., 2023), whose ERG response amplitudes reached negative ERG thresholds around PN 8-mo (DA) and PN 2-mo (LA), response amplitudes of T206A/T206A mice did not reach the negative ERG threshold under either DA or LA conditions out to PN 12-mo. However, the compound heterozygous T206A/K42E mice met the negative ERG criterion under DA (1.0±0.1) and LA conditions (0.8±0.1) at PN 12-mo.

Intensity-response data, which show the relationship between the intensity of stimuli and the resulting amplitude response from rods or cones (Breton et al., 1991), were compared. At PN 1-mo, the ERG parameter rod R_max_ (i.e. the saturating b-wave amplitude) values for T206A/T206A and T206A/K42E mice (377.9±38.9 µV and 392.7±53.8 µV, respectively) were comparable to that of WT mice (438.4±40.0 µV). However, at PN 12-mo R_max_ values were significantly lower (*P*≤0.05) for T206A/T206A and T206A/K42E mice (117.1±23.2 µV and 109.7±32.0 µV, respectively) than for WT mice (274±31.6 µV). LA R_max_ (cone-driven responses to the same 6.955 log photons/µM^2^ flash) could, however, not be determined because a photopic intensity response series had not been recorded.

Implicit time (or time-to-peak (TTP) was measured for a- and b-waves under DA (Fig. 4E) and LA (Fig. 4F) conditions for WT, T206A/WT, T206A/T206A and T206A/K42E mice. Under DA conditions, the a-wave time-to-peak value for T206A/T206A and T206A/K42E mice differed significantly from those of WT mice at PN 1-mo but, surprisingly, did not differ compared to those of WT mice at PN 12-mo. As previously reported for K42E/K42E mice (Nguyen et al., 2023), b-wave time-to-peak values for mutant animals under DA conditions, have significantly higher implicit times compared to those of WT mice at PN 1-mo or PN 12-mo. However, under LA conditions, no implicit times did not differ significantly to those of WT mice at PN 1-mo or PN 12-mo (Nguyen et al., 2023).

Oscillatory potentials (OP) or small wavelets on the rising phase of the b-wave were analyzed by calculating the area under the curves for WT, K42E/K42E, T206A/T206A and T206A/K42E mice. Graphs of averaged OPs under LA and DA conditions are shown in Fig. 4G and H, respectively. All *Dhdds* mutants had significantly lower OP wave amplitudes (≥40% reduction) compared to those of WT.

### Reduced c- and d-wave amplitudes indicate inner retinal dysfunction

**Fig. 5.**
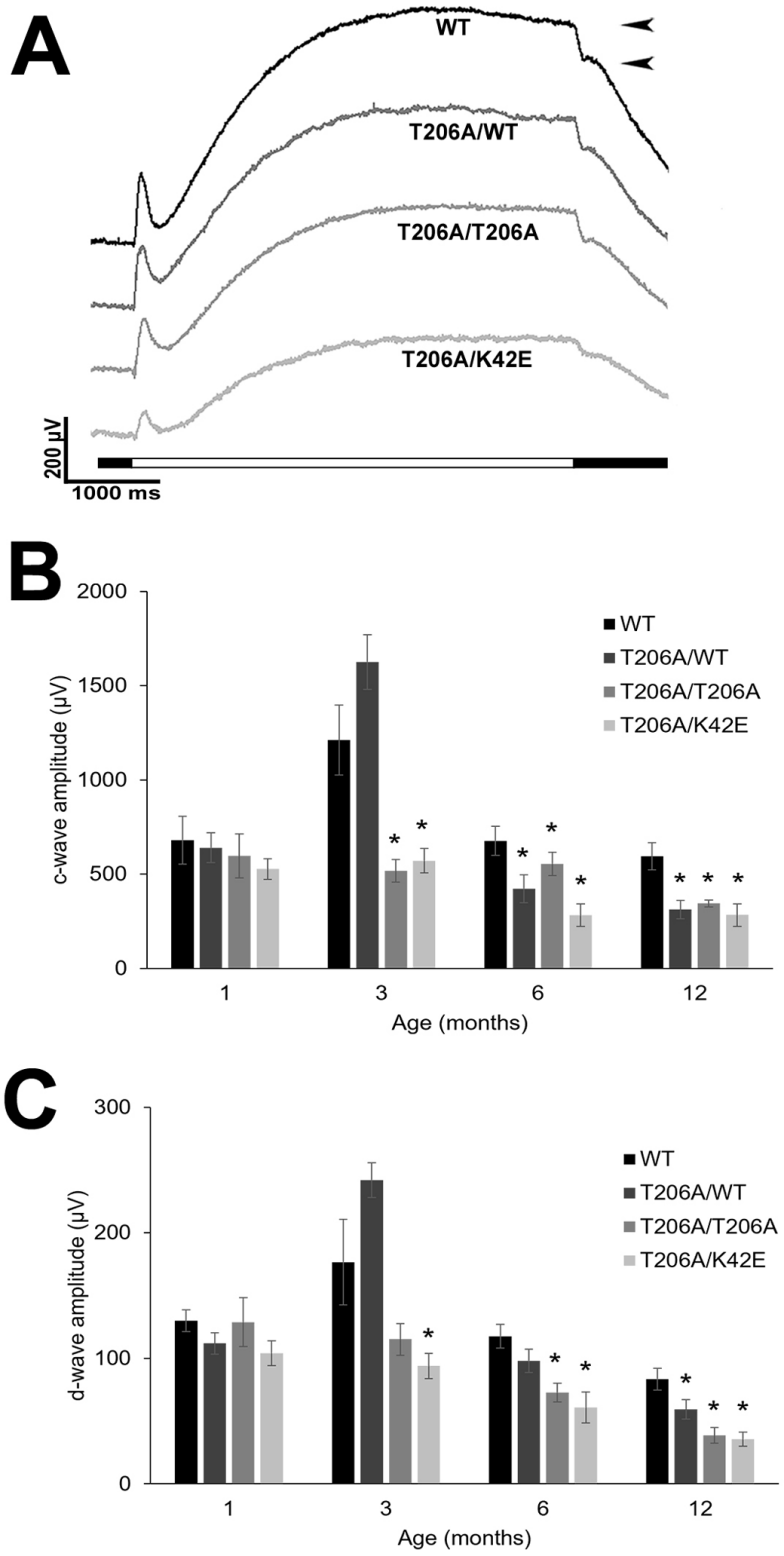
**ERG c- and d-wave amplitudes.** (A) Representative PN 6-mo waveforms for c- and d-waves are shown. Black arrowheads indicate measurement of d-wave amplitudes; horizontal bar indicates 5000 ms stimulus on (white bar) and stimulus off (black bar). (B,C) Plotted are measurements of c-wave amplitudes (B) and d-wave amplitudes (C) at PN 1-, 3-, 6-, and 12-mo for mice as indicated compared to those of WT mice. Statistical significance: *P≤0.05. c-waves, (WT, T206A/WT, T206A/T206A, T206A/K42E): PN 1-mo (*n*=9, 8, 8, 8); PN 3-mo (*n*=9, 9, 9, 8); PN 6-mo (*n*=7, 8, 8, 6); PN 12-mo (*n*=8, 7, 7, 8); d-waves: PN 1-mo (*n*=8, 8, 8, 8); PN 3-mo (*n*=9, 9, 9, 9); PN 6-mo (*n*=7, 8, 8, 7); PN 12-mo (*n*=8, 7, 8, 8).

Amplitudes of the ERG c-wave, which represent RPE and Müller cell contributions (Steinberg, 1985; Zeumer et al., 1994), were analyzed from PN 1-mo to PN 12-mo. Representative PN 6-mo waveforms are shown in Fig. 5A. The c-wave amplitudes were averaged and graphed to compare each mutant mouse line against WT mice for each time point (Fig. 5B). At PN 1-mo, c-wave amplitudes did not differ across the genotypes. At PN 3-mo, c-wave amplitudes for T206A/T206A and T206A/K42E mice were significantly lower than those recorded in WT mice (*P*≤0.05). In contrast, the c-wave amplitudes for T206A/WT mice were not significantly different from those of WT mice. At both PN 6- and 12-mo, c-wave amplitudes for all mutant genotypes were significantly lower than those of WT (*P*≤0.05).

ERG d-wave amplitudes (Fig. 5A, see arrowheads,) were measured and analyzed (Fig. 5C) to assess the response of the OFF-cone bipolar cells (CBCs) (Naarendorp and Williams, 1999). Similar to c-wave analysis at PN 1-mo, d-wave amplitudes for mice of all mutant genotypes showed no differences compared to age-matched WT mice. However, at PN 3-mo, d-wave amplitudes of T206A/K42E mice were reduced by 46.8% compared to those of age-matched WT mice (*P*≤0.05). By PN 6-mo, d-wave amplitudes of both T206A/T206A and T206A/K42E mice also were significantly reduced (38% and 48.3, respectively, *P*≤0.05) compared to WT, and by PN 12-mo, mice of all mutant genotypes showed reduced d-wave amplitudes (T206A/WT, 29%; T206A/K42E, 57.3%; T206A/T206A, 53.8%; all *P*≤0.05) compared to WT.

### All *Dhdds* mutants show significant inner retina cell reductions

**Fig. 6.**
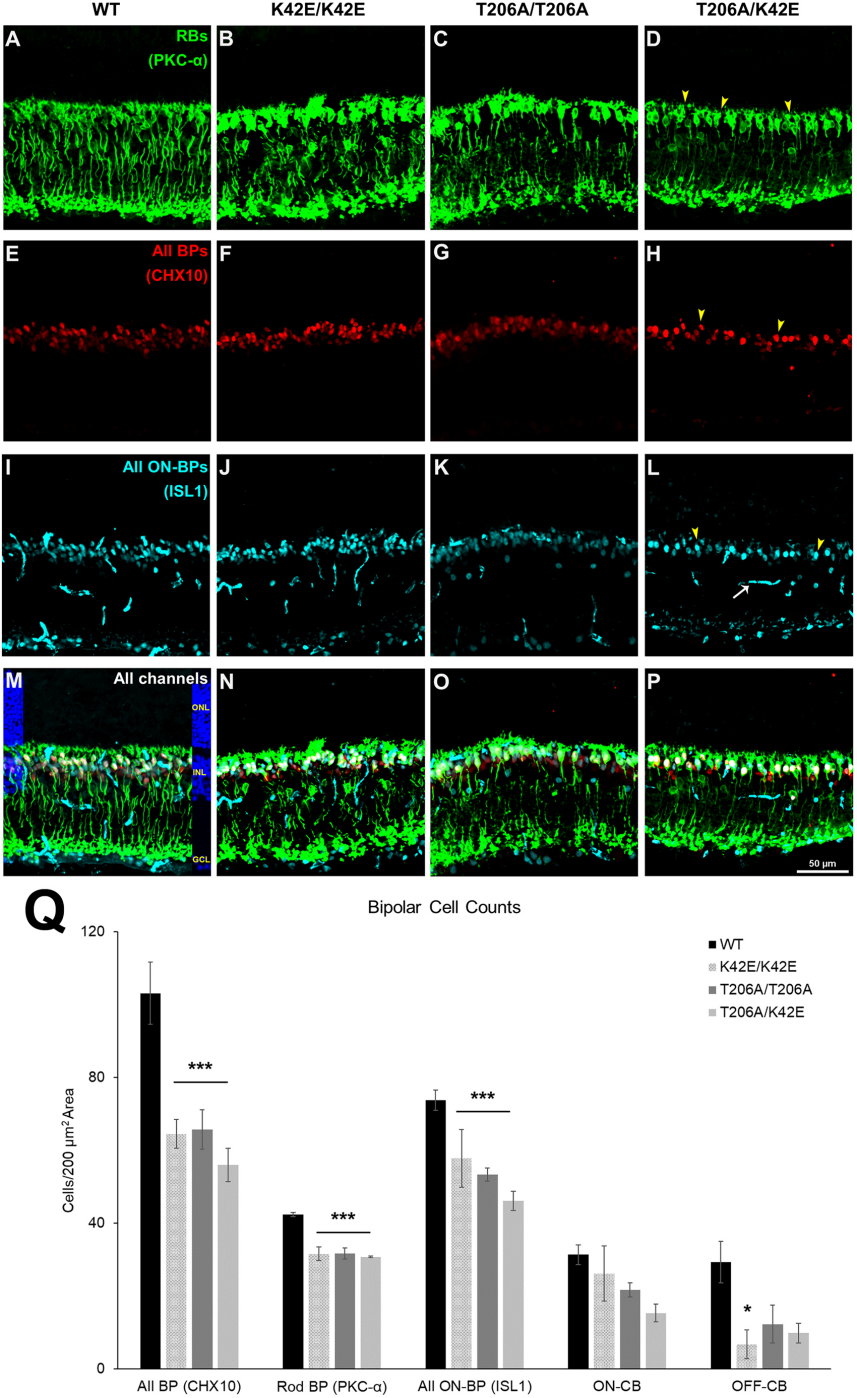
**Bipolar cell densities.** (A-P) Shown are representative 200 µm×200 µm images of retinal tissue sections obtained from WT (column 1, panels A,E,I,M), K42E/K42E (column 2, panels B,F,J,N), T206A/T206A (column 3, panels C,G,K,O) or T206A/K42E (column 4, panels, D,H,L,P) mice. Rod bipolar cells (RBs) labeled with PKC-α are shown in green (A-D). All bipolar cells (All BPs) labeled with anti-CHX10 are shown in red (E-H). All-ON- bipolar cells (All ON-BCs) labeled with anti-ISL1 are shown in cyan (I-L). Merged images are shown in panels M-P; the DAPI panel overlay (blue) in M indicates the nuclear layers of the retina. Yellow arrowheads (D,H,L) represent puncta that were counted. White arrow in panel L indicates ISL1-positive blood vessels, which were excluded from bipolar cell counts. (Q) Averaged overall values for All BP, Rod BP, All ON-BP, ON-CB and OFF-CB cell counts per 200 µm×200 µm squares are plotted. RPE, retinal pigment epithelium; OS, outer segment layer; IS, inner segment layer; ONL, outer nuclear layer; OPL, outer plexiform layer; INL, inner nuclear layer; IPL, inner plexiform layer; GCL; ganglion cell layer. Scale bar: 50 µm for all panels. Statistical significance: *P≤0.05 and ***P≤0.001. *n*=3 for WT, K42E/K42E, T206A/T206A and T206A/K42E.

Because retinas of all mutant mice analyzed by ERG, morphologic and SD-OCT showed alterations consistent with inner retina compromises, we further explored structural changes in the inner retina of ≥PN 8-mo mutant mice (Fig. 6). Triple immunolabeling with inner retina cell type-specific antibodies – against CHX10 (officially known as VSX2) to identify all bipolar (BC) cells, against ISL1 to identify ON-bipolar cells or against PKC-α (officially known as PKCA) to identify rod bipolar cells (RBCs) (see also Table 1) – were used to assess the integrity of these cell populations. For analysis, bipolar cells were classified by immunolabeling signature in each *Dhdds* mutant line and compared to WT controls to assess the effect of the different *Dhdds* mutations on RBCs, ON-CBCs and OFF-CBCs (Fig. 6Q).

**Table 1.** Antibodies

Antibody	Host	Dilution	Vendor, catalog no.	RRID
Chx10	Sheep, polyclonal	1:200	GeneTex, GTX16142	AB_368709
ISL1	Mouse, monoclonal	1:100	Developmental Studies Hybridoma Bank, 39.4D5	AB_2314683
PKC-α	Rabbit, polyclonal	1:200	Abclonal, A0267	AB_2757080
MEIS2	Mouse, monoclonal	Undiluted	Developmental Studies Hybridoma Bank, 2B4	AB_2618844
TCF4	Rabbit, polyclonal	1:150	Proteintech, 22337-1-AP	AB_2879076
Donkey anti-Sheep Alexa Fluor 647	Donkey/IgG	1:500	Invitrogen, A21448	AB_2535865
Donkey anti-Mouse Alexa Fluor 488	Donkey/IgG	1:500	Invitrogen, A21202	AB_141607
Donkey anti-Rabbit Alexa Fluor 546	Donkey/IgG	1:500	Invitrogen, A10040	AB_2534016

Analysis of total CHX10-positive labeling showed significant bipolar cell loss in all three *Dhdds* mutant strains compared to WT mice (K42E/K42E, 36% reduction; T206A/T206A, 36% reduction; T206A/K42E, 43% reduction; *P*≤0.001 for all comparisons) (Fig. 6Q). Analysis of PKC-α immunolabeling revealed significant loss of RBCs in all *Dhdds* mutant lines compared to WT mice (K42E/K42E, 29% reduction; T206A/T206A, 23% reduction; T206A/K42E, 27% reduction. *P*≤0.001 for all comparisons, Fig. 6Q).

To analyze the effects of the various *Dhdds* mutations on the ON-CBC population, i.e. to determine the number of ON-CBCs for each mouse strain, we subtracted the number of PKC-α-positive RBCs from the total number of ISL1-positive ON-bipolar cells in each specimen. We found a reduction of ON-CBCs in each *Dhdds* mutant strain no statistically significant differences compared with ON-CBCs in WT mice: K42E/K42E, 16% reduction; T206A/T206A, 31% reduction; T206A/K42E, 51% reduction compared to WT (Fig. 6Q). To determine whether *Dhdds* mutations affected the population of OFF-CBCs, the total number of ISL1-positive bipolar cells (RBC-positive ON-CBCs) was subtracted from the total number of bipolar cells (Chx10-positive cells) in each specimen from each *Dhdds* mutant mouse strain and compared to WT controls. Reductions in OFF-CBCs were: 77% for K42E/K42E, 58% for T206A/T206A and 66% for T206A/K42E mice, with significantly reduced OFF-CBCs numbers compared to those in WT mice only in retinas of K42E/K42E mice (*P*≤0.05).

**Fig. 7.**
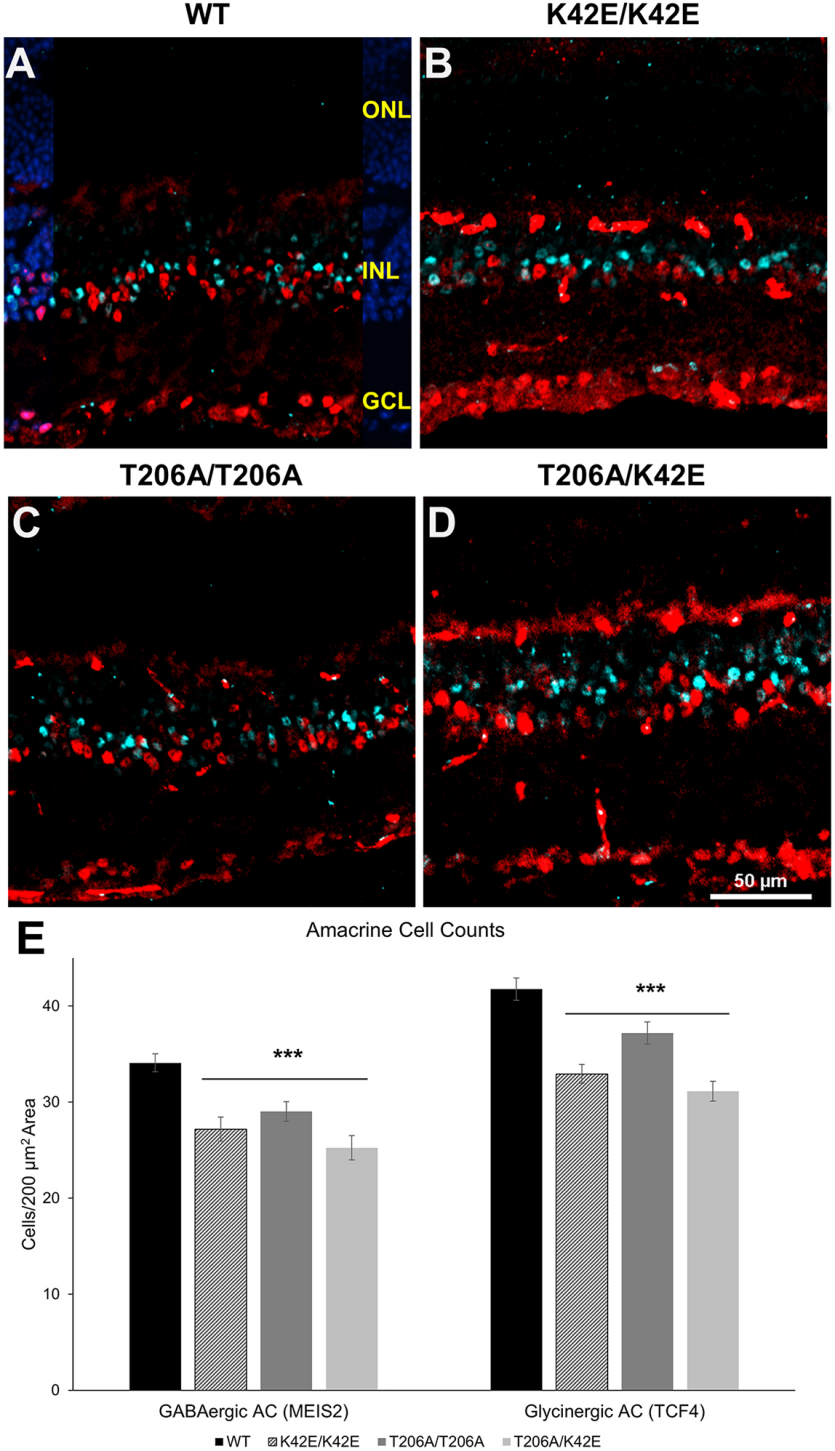
**Amacrine cell densities.** (A-D) Representative images of immunolabeled retinal tissue sections obtained from WT (A), K42E/K42E (B), T206A/T206A (C) and T206A/K42E (D) mice. GABAergic amacrine cell (AC) nuclei show immunolabeling for MEIS2 (pseudo-colored in red), glycinergic AC nuclei show immunolabeling for TCF4 (pseudo-colored in cyan). The DAPI (blue) panel overlay in A indicates the nuclear layers of the retina. (E) Averaged overall values for GABAergic and glycinergic AC nuclei counts are plotted. ONL, outer nuclear layer; INL, inner nuclear layer; GCL; ganglion cell layer. Scale bar: 50 µm for all panels. Statistical significance: ***P≤0.001. *n*=3 for WT, K42E/K42E, T206A/T206A and T206A/K42E.

Given the thinning of the INL and reduced bipolar cell populations in *Dhdds* mutants, we also examined the amacrine cell (AC) population. To identify ACs, we used immunostaining against MEIS2, a transcription factor expressed in GABAergic amacrine cells, and TCF4, a transcription factor expressed in glycinergic amacrine cells (Yan et al., 2020). Representative double immunofluorescence staining of MEIS2 and TCF4 is shown in Fig. 7A-D, with MEIS2 pseudo-colored in red and TCF4 pseudo-colored in green. Cells were counted in the same manner as bipolar cells. Averaged GABAergic and glycinergic AC populations for each genotype were plotted and are shown in Fig. 7E. All *Dhdds* mutants, i.e. K42E/K42E, T206A/T206A and T206A/K42E, had reduced numbers of GABAergic and glycinergic AC compared to age-matched WT mice. T206A/K42E mice had the largest difference compared to WT with a 25% AC loss (*P*≤0.001) in both AC types; K42E/K42E showed a 20% AC loss (*P*≤0.001). In comparison, the T206A/T206A mutants showed a less severe AC loss with a 10% dropout of glycinergic ACs and a 14% loss of GABAergic ACs (*P*≤0.001 for both).

### Visual acuity is significantly reduced in *Dhdds* mutant mice relative to controls

**Fig. 8.**
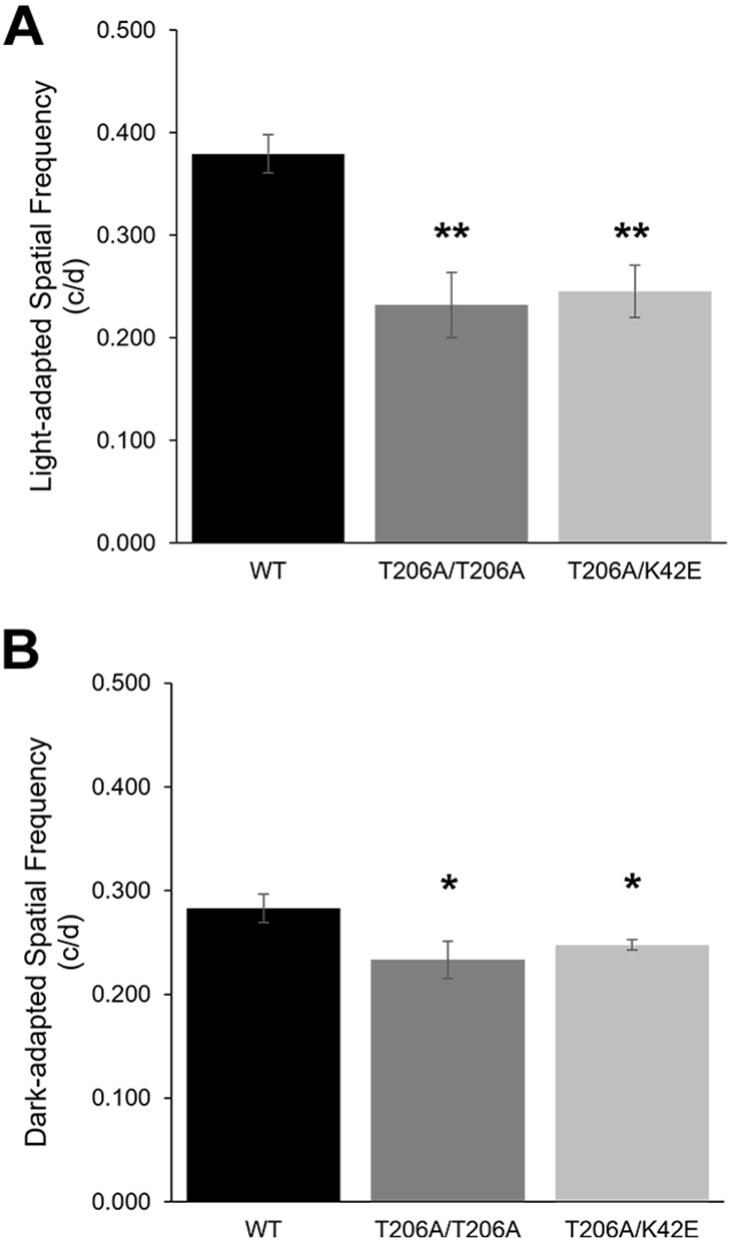
**Visual acuity values at** PN **12-mo.** (A,B) The optokinetic response was used to determine the highest spatial frequency and visual acuity for WT, T206A/T206A, and T206A/K42E mice at PN 12-mo under light-adapted (A) or dark-adapted (B) conditions. c/d, cycles per degree. Statistical significance: *P≤0.05 and **P≤0.01. *n*≤3 for WT, T206A/T206A and T206A/K42E.

Optokinetic response (OKR) experiments in WT, T206A/T206A and T206A/K42E mice were performed at PN 12-mo to assess the status of signal transmission from the retina to the brain. Spatial frequency was measured, highest values were recorded and visual acuity values were plotted under LA (Fig. 8A) or DA conditions (Fig. 8B). Compared to WT controls, T206A/T206A and T206A/K42E mice both showed significantly lower visual acuity under both LA and DA conditions (*P*≤0.05).

## DISCUSSION

We generated and characterized a novel mouse line containing one of the disease-associated *DHDDS* variants found in human RP59 patients. Here, we aimed to create a tractable and informative RP59 animal model to better understand the underlying mechanism leading to the human disease. Previously, we have studied the K42E/K42E knock-in mouse model based upon the primary *DHDDS* variant found in RP59 patients, where we found reduced total retinal and INL thicknesses, photoreceptor terminal retraction, second-order neuronal sprouting, ectopic cell displacement, and negative ERG waveforms without any evidence of defective protein N-glycosylation (Nguyen et al., 2023; Ramachandra Rao et al., 2020). In our present study, relevant to T206A, the second most-prominent *DHDDS* variation found in RP59 patients, we also assessed the structural and functional effects of the variant on the retina. As hypothesized, T206A/K42E knock-in mice expressed a comparable phenotype to that of K42E/K42E knock-in mice. Surprisingly, studied independent from the K42E allele, mice with the T206A/T206A variant that has not been found in humans, also showed similar phenotypes. T206A/T206A knock-in mice showed declining structural and functional phenotypes with age but, most importantly, also showed a reduction in bipolar cell density, indicating increased bipolar cell sensitivity to insult as a result of DHDDS mutation.

As expected with retinal aging, total retinal thickness was reduced at PN 12-mo but the INL thickness in T206A/T206A and T206A/K42E mice showed significant reductions of 26% and 30%, respectively (compared to 11% in age-matched WT mice, Fig. 3). Histological analysis of retinal tissue sections performed on younger animals (PN≤6-mo) showed no compromise of gross retinal structure in T206A/T206A or T206A/K42E mice. However, older T206A/T206A and T206A/K42E mice (PN≥6-mo) exhibited reduced total retinal thickness as well as displacement of photoreceptor cells from the ONL (*arrows*, Fig. 2). Previously published studies using K42E/K42E mice showed increased GFAP immunolabeling, increased cell death (TUNEL staining), and photoreceptor terminal retraction and sprouting of RBC dendrites into the ONL (Nguyen et al., 2023; Ramachandra Rao et al., 2020). However, there were no significant changes in ONL thickness, indicating that the aforementioned histological changes are not merely due to increasing age and are restricted to the *inner* retina rather than the photoreceptor layer. The T206A allele has a milder degenerative phenotype than the K42E allele and, in mice, both alleles are less severe than that reported for RP59 patients (Kimchi et al., 2018). Many phenotypes found in these patients point to issues in and around the fovea, and many of the RP59 patients are misdiagnosed with cone-rod dystrophy instead of RP59. Furthermore, we have recently reported an associated modifier gene polymorphism (ALG6 F304S) that correlates with increased severity of macular cone disease and reduced severity of peripheral rod disease (Monson et al., 2024). Thus, altered macular structure/function clearly has a significant role in RP59 pathology; however, this aspect of the disease cannot be modeled by using mice, as mouse retina lacks a macula. That said, *Dhdds* mouse models remain valuable with respect to understanding the inner retina changes caused by *Dhdds* mutations that have significant impact on the disease and, due to the inherent limitations of human studies, have not been recognized in RP59 patients.

Because of histological thinning of the INL and evidence of inefficient synaptic transmission to second-order neurons evidenced by ERG, we used the BC-specific anti-CHX10 antibody and immunofluorescence imaging to identify BCs. The cell bodies of BCs make up 40-50% of the INL (Wässle et al., 2009). Upon visual inspection, it was apparent that there were fewer CHX10-positive cells in mutant compared to WT retina (Fig. 6E-H). Overall, compared to WT, all mutants had fewer BCs per 200 µm×200 µm square, with T206A/K42E mice showing the biggest reduction with 46%. The most fascinating piece of data we found from BC density analysis was the significant (and selective) reduction of ON-BCs (depicted by PKC-α-positive and ISL1-positive cells, Fig. 6), which correlates directly to the observed negative ERG (Fig. 4). The reduced b-wave amplitudes and decreased sensitivity under DA conditions (Fig. 4C and E; Table S1) correlate directly to the significantly reduced RBCs densities, as the b-wave is initiated from ON-BCs (Bhatt et al., 2023; Creel, 2019). While mutant rod BC counts were reduced compared to those of WT, ON-CBC counts were not. Although we did not probe directly for OFF-CBCs, we calculated estimates by taking the numbers of ISL1-positive cells and subtracted from the total number of CHX10-positive cells, which revealed a marked and significant reduction in K42E/K42E OFF-CBCs (77% fewer than WT, Fig. 6Q). However, T206A/T206A and T206A/K42E OFF-CBC counts were not statistically lower than those for WT. Reduced OFF-CBCs are in agreement with the reduced d-wave ERG amplitudes found in mutant mice (Fig. 5) (Perlman, 2007). That said, at present we do not have a definitive mechanism to explain why the drop-out of bipolar cells, as evidenced by morphometric (histological) data and functional (ERG) data.

Because the inner retina is very thin in older *Dhdds* mutant mice, (see Figs 2 and 3), loss of amacrine cells was expected (Fig. 7). It is estimated that ∼50% of amacrine cells in mammalian retinas are GABAergic, and that ∼40% of amacrine cells are glycinergic and critical to processing of signals transmitted to ganglion cells (Davanger et al., 1991; Perez-Leon et al., 2022). Therefore, labeling GABAergic and glycinergic amacrine cells would identify ∼90% of the total AC population. All *Dhdds* mutant strains showed reduced AC density, consistent with the reduction in bipolar cell density. Further, these data are congruent with the reduced oscillatory potentials (Fig. 4G,H). Oscillatory potentials arise from inhibitory pathways mediated by GABAergic and glycinergic ACs in the IPL (Wachtmeister, 1998). Thus, the reduced oscillatory potential observed in this study suggests that loss of GABAergic and glycinergic ACs in *Dhdds* mutant mice significantly impairs synaptic processing by amacrine cells in the IPL.

To test whether T206A *DHDDS* variants caused dysfunction in the transmission of light-driven visual signals to the brain, we performed OKR experiments on PN 12-mo mice (Fig. 8). Unlike humans, mice have laterally positioned eyes and lack the ability to rotate their eyes. OKR – which is performed in awake and unrestrained animals, allowing head movement – provides an accurate, quantitative method to assess visual competence, as represented by calculation of numerical values for visual acuity from spatial frequency measurements or the ability to resolve two points in space (Stahl, 2004). Through these experiments, we found that T206A/T206A and T206A/K42E knock-in mouse lines both have reduced visual acuity (i.e. greater spatial frequency values), compared to age-matched WT mice. Compared to previously published OKR data (Nguyen et al., 2023), K42E/K42E mice had lower LA visual acuity than T206A/K42E and T206A/T206A mice. We suspect that loss of bipolar cells and impaired transmission of signals through the inner retina arising from the K42E and T206A mutations together impair the visual performance of our *Dhdds* mutant mice.

As shown previously, K42E/K42E *Dhdds* mutant mice display a negative ERG as early as PN 2-mo (Nguyen et al., 2023). In RP59 patients, the T206A *DHDDS* variant has, to date, only been found heterozygously with the K42E variant (Wen et al., 2013). The K42E/K42E *Dhdds* mutation caused a severe phenotype in the knock-in mice, while T206A/K42E mutants exhibited a similar but less severe phenotype. Apparently, residue K42, which is in direct contact with the *cis*-prenyltransferase active site, is particularly important for the activity of the protein (Bar-El et al., 2020; Lisnyansky Bar-El et al., 2019). As found in patients and in the K42E/K42E mouse model, the K42E variant led to shortened dolichol species (Nguyen et al., 2023; Wen et al., 2013). The hypothesis that the K-to-E change from a positively to a negatively charged amino acid mutation hinders the active site of the *cis*-prenyltransferase protein is supported by the more severe phenotype in K42E/K42E mice and the less severe phenotype in our T206A/K42E mice (Lisnyansky Bar-El et al., 2019). An even milder phenotype is shown in T206A/T206A mice, possibly because T206 perturbs the active site as its threonine backbone is hydrogen-bound to the backbone of the metal-binding aspartate residue at position 34 – as hypothesized previously (Edani et al., 2020). However, the question of why the mutations lead to non-syndromic RP remains unresolved.

In summary, the results presented here – achieved by the characterization of our novel mouse models harboring the T206A *Dhdds* mutation – provide some informative insights, by extension, about the human disease RP59. Because T206A/K42E is one of the prevalent variants reported in RP59 patients, these findings will bring us closer to understanding the mechanism underlying this disease.

## MATERIALS AND METHODS

### Animals

*Dhdds*^T206A/WT^ mice were created on a C57Bl/6J background by the UAB Heflin Genomics Center using CRISPR-Cas9 technology. Briefly, CRISPR guides were designed using CRISPOR (http://crispor.tefor.net) to target exon 7 in the mouse *Dhdds* locus using the following amplicon (see Materials and Methods section: PCR genotyping) with a silent DNA polymorphism to eliminate the PAM recognition site downstream of the target site for DNA cleavage (Anders et al., 2014). Founder animals were identified by PCR, by using primers flanking the target loci to amplify a 399 base pair fragment in wild-type (WT) animals.

Sequence-verified heterozygous mice were crossed to create WT and homozygous *Dhdds*^T206A/T206A^ mice. Homozygous *Dhdds* T206A/T206A mice were crossed with homozygous *Dhdds* K42E/K42E mice to create compound heterozygous T206A/K42E mice. The genotype of cross-bred mice was confirmed by PCR and DNA sequencing (see Materials and Methods section: PCR genotyping). All procedures conformed to the ARVO Statement for the Use of Animals in Ophthalmic and Vision Research and were approved by the Institutional Animal Care and Use Committee (IACUC) of the University of Alabama at Birmingham.

Animals were maintained on a standard 12 h/12 h light/dark cycle (20–40 lux ambient room illumination), fed standard rodent chow, provided water *ad libitum* and were housed in plastic cages with standard rodent bedding.

### PCR genotyping

Initial genotype verification was completed at the UAB Heflin Center for Genomic Sciences Core Laboratories, validating the heterozygous T206A/WT strain. PCR primers were purchased from Invitrogen (Waltham, MA, USA) to validate tail snip DNA sequences: (forward primer: 5′-TGGGTGATCTGCATCTGCTG-3′; reverse primer: 5′-GTGCACCATGGTTCCTCTGA-3′). DNA sequences were confirmed in subsequent generations by digestion with restriction enzymes *Bcl*I (for T206A) and *Sty*I (for K42E) that cleave the respective knock-in alleles.

The WT amplicon used was: 5′-TGGGTGATCTGCATCTGCTGCCCTTGGACCTCCAGGAGAAGATTGCGCATGCCATCCAGGCTACTAAGAACTACAATAAGTGTTTCCTCAATGTCTGCTTTGCATACACATCACGTCATGAGATTGCCAATGCTGTGAGAGAGATGGCCTGGGGCGTGGAACAAGGTCTGCTGGAACCCAGTGATGTCTCCGAGTCTCTGCTCGATAAGTGCCTCTATAGCAACCACTCTCCTCATCCCGACATCCTGATCCGG**ACT**TCTGGGGAGGTGCGGCTGAGTGACTTCTTGCTCTGGCAGACGTCCCATTCCTGCCTCGTGTTCCAGCCTGTCCTGTGGCCAGAATACACATTTTGGAACCTGTGTGAGGCAATTCTGCAGTTTCAGAGGAACCATGGTGCAC-3′; nucleotides encoding the threonine residue at position 206 (T206) are shown in bold. The 200 bp single-stranded DNA oligonucleotide repair template used was: 5′-CTGTGCTTCTGTCTCCTGCCCACCTAGTGATGTCTCCGAGTCTCTGCTCGATAAGTGCCTCTATAGCAACCACTCTCCTCATCCCGACATCCTGATC*aGG**gCT**TCTGGGGAGGTGCGGCTGAGTGACTTCTTGCTCTGGCAGGTAGGTTGTTTTGAAACATGTTATTTTGGGGTTGGGCTGAACCCTGGAACTGAAGCAG-3′. Nucleotides encoding the alanine residue at position 206 (A206) are shown in bold; the *Bcl*I restriction site unique to the mutant allele is shown in italics; lowercase letters indicate unique sequences in the T206A variant construct; the asterisk (*) indicates the CGG→AGG silent mutation at the CRISPR PAM recognition site.

### Spectral domain optical coherence tomography

*In vivo* retinal imaging was performed using a Bioptigen Model 840 Envisu Class-R high-resolution spectral domain optical coherence tomography (SD-OCT) instrument (Bioptigen/Leica, Inc), as described previously by DeRamus et al. (2017). The retinal layer thickness was measured using the Bioptigen (Leica, Inc.) Diver V 3.4.4 software and spot-checked manually using calipers on the Bioptigen (Leica, Inc.) InVivoVue software. Data were collected from at least *n*=3 independent WT, T206A/T206A, T206A/WT and T206A/K42E mice at PN 1-mo and PN 12-mo.

### Optokinetic response

To assess how the brain is responding to visual stimuli, the optokinetic response (OKR) was measured as described by Gil et al. (2022) under scotopic [dark-adapted (DA)] and photopic [light-adapted (LA)] conditions using an OptoMotry HD instrument (Cerebral Mechanics Inc.). Data were analyzed using Microsoft 365 Excel and visualized graphically.

### Electroretinography (ERG)

Mice were DA overnight and ERGs were recorded on a custom-built system as described previously (Nguyen et al., 2023). Briefly, ERG a- and b- wave responses were recorded following 2-ms flashes of light with increasing flash intensities under DA and LA conditions. ERG c- and d- waves were recorded following a 5-s step of light stimulus by using the DC recording mode. Implicit times for a- and b- wave responses were recorded at the highest intensities, i.e. the brightest flash. R_max_ was calculated and averaged after plotting intensity-response curves within the Igor Pro software WaveMetrics, Inc., Portland, OR, USA; https://www.wavemetrics.com/.

Oscillatory potentials were analyzed for waves recorded following the highest light stimuli (6.955 log photons/µM^2^) under DA and LA conditions. Methods were as described by Hancock and Kraft (2004). Briefly, a high-pass filter of 70 Hz and a low-pass filter of 34 Hz were applied to each wave. The area under the curve between *time*=0 s and *time*=0.15 s was calculated, and noise was subtracted to quantify the amplitudes of oscillatory potentials.

Response amplitudes were averaged using Data Browser V 6.4.4 software (LabVIEW by National Instruments, Austin, TX, USA) and analyzed with Igor Pro 8 and 9.

### Light microscopy (histology)

Mouse eyes were enucleated following euthanasia and cervical dislocation, fixed in buffered mixed aldehydes and processed for Epon 812 resin embedment as described previously (Sarfare et al., 2014). Retinal tissue sections (0.8-µm thickness) were obtained using a microtome, collected on glass microscope slides and stained with 0.1% Toluidine Blue. Histological images were collected using an Olympus VS-120 photomicroscope (BX61VS platform) running Olympus VS-ASW-2.9 software.

### Immunofluorescence

Mouse eyes were processed to obtain retinal cryosections as previously described (Wang et al., 2017). In brief, after euthanasia and enucleation, eyes were fixed by immersion in chilled 4% paraformaldehyde (PFA) in 0.1 M PBS for 15 min and the anterior segments were removed. The resulting eyecups were cryoprotected by serial immersion in graded buffered sucrose solution, and then embedded and frozen in optimal cutting temperature compound. Cryosections (10-12 µm thickness) were obtained using a cryotome and collected on glass microscope slides, rehydrated in PBS and blocked using 10% donkey serum in PBS containing 0.1% Triton X-100 (PBST) for 1 h, before being incubated with primary and appropriate fluorescent secondary antibodies (Alexa Fluor 647, 488, 546; see Table 1 for antibody information). Following counterstaining nuclei with DAPI coverslips were added onto slides. Eyes from *n*=3 different animals per genotype (WT, *Dhdds*^K42E/K42E^, *Dhdds*^T206A/T206A^, and *Dhdds*^T206A/K42E^) between age ranges PN 8-mo to PN 12-mo were used. At least *n*=3 retinal sections (including or proximal to the optic nerve head) were subjected to immunofluorescence microscopy analysis per genotype and age group, and at least *n*=5 images (200 µm×200 µm) acquired from both sides of the optic nerve head were analyzed from each retinal section.

Labeled retinal cryosections were imaged using a Nikon AX-R confocal microscope at 20× magnification. *Z*-stack images were compressed, background noise was removed and images were analyzed using the NIS-Elements AR imaging software (Nikon Instruments, version 5.21.03). Labeled cell bodies within each 200 µm×200 µm square were counted manually in each fluorescence channel and catalogued in the NIS-Elements Object Count tool. Similarly sized cell nuclei (Fig. 6, indicated by yellow arrows) were counted and catalogued, while smaller or larger-than-average nuclei were excluded to focus counting on puncta within the same surface plane. Cell counts were averaged and analyzed using R software (version 4.2.1).

### Statistical analysis

Statistical analysis was done using two-tailed Student's *t*-test assuming equal variances or, alternatively, using ANOVA with post-hoc Tukey's test to compare quantitative data obtained from T206A/T206A, T206A/WT and T206A/K42E mice in comparison with age-matched WT mice. Outlier values were identified and removed using the interquartile range method. Statistical significance *P*-value thresholds were: *P<0.05, **P<0.01 and ***P≤0.001. For all experiments the error reported is the ± standard error of the mean (±s.e.m.).

## Supplementary Material

10.1242/dmm.052243_sup1 Supplementary information
