# Supplementary material for: Dhdds T206A and Dhdds K42E knock-in mouse models of retinitis pigmentosa 59 are phenotypically similar
Source: Dis Model Mech. 2025 Aug 1;18(7):dmm052243. doi: 10.1242/dmm.052243 (PMC12352288; doi:10.1242/dmm.052243)
Supplement: Supplementary information [file dmm-18-052243-s1.pdf]

Table S1. ERG Raw Data. Raw, averaged b-wave amplitudes, a-wave amplitudes, sample number n, and b/a ratios ± standard error of WT, T206A/WT, T206A/T206A, and T206A/K42E animals at PN 1-, 3-, 6-, and 12-mo. Statistical significance comparing mutant to age-matched WT: $^{1}p \leq 0.05$, $^{2}p \leq 0.01$, and $^{3}p \leq 0.0001$.

Dark-adapted ERG Responses

	WT				T206A/WT				T206A/T206A				T206A/K42E			
age (mo)	b-wave	a-wave	n	b/a ratio	b-wave	a-wave	n	b/a ratio	b-wave	a-wave	n	b/a ratio	b-wave	a-wave	n	b/a ratio
1	712±96	367±44	9	1.9±0.1	620±60	324±35	8	1.9±0.1	614±86	340±46	7	1.8±0.1	560±73	337±42	8	1.7±0[2]
3	760±79	402±35	10	1.8±0	1014±106[1]	497±58	8	2.0±0.1[2]	493±21[2]	313±8[1]	8	1.6±0[3]	452±40[2]	335±28	8	1.3±0[3]
6	566±75	320±31	8	1.8±0.1	451±28	245±14[1]	8	1.8±0[1]	403±28[1]	283±24	8	1.4±0[3]	313±40[1]	264±33	8	1.2±0[3]
12	415±29	265±21	8	1.6±0.1	258±33[2]	159±22[2]	7	1.6±0	203±30[3]	167±19[2]	8	1.2±0.1[3]	180±18[3]	184±24[1]	8	1±0.1[3]

Light-adapted ERG Responses

	WT				T206A/WT				T206A/T206A				T206A/K42E			
age (mo)	b-wave	a-wave	n	b/a ratio	b-wave	a-wave	n	b/a ratio	b-wave	a-wave	n	b/a ratio	b-wave	a-wave	n	b/a ratio
1	318±36	174±23	9	1.8±0.2	304±30	178±20	7	1.7±0	231±27	137±20	8	1.7±0.2	322±63	191±28	7	1.7±0.1
3	286±32	174±20	10	1.6±0.1	394±25[1]	228±17	9	1.7±0.1	239±32	173±21	11	1.4±0.1[2]	199±22[1]	172±16	9	1.2±0.1[2]
6	198±25	135±10	8	1.5±0.1	155±21	82±14[3]	7	1.9±0.3[1]	108±6[1]	108±10	6	1.0±0.1[1]	115±17[1]	121±13	6	1.0±0.1[3]
12	183±12	124±10	8	1.5±0.1	134±15[1]	81±10[1]	8	1.7±0.1	108±14[3]	106±12	8	1.0±0.1[2]	83±13[3]	101±9	8	0.8±0.1[3]
